# Supplementary material for: A counsellor-supported ‘PTSD Coach’ intervention versus enhanced Treatment-as-Usual in a resource-constrained setting: A randomised controlled trial
Source: Glob Ment Health (Camb). 2024 Jan 3;11:e7. doi: 10.1017/gmh.2023.92 (PMC10808979; doi:10.1017/gmh.2023.92)
Supplement: Bröcker et al. supplementary material 1 — Bröcker et al. supplementary material [file S2054425123000924sup001.docx]

Supplementary tables:

*Supplementary Table 1. PTSD Coach Survey scores (n = 27).*

| **Item** | **Item** | **Mean (SD)** |
| --- | --- | --- |
| 1. | Helping me learn about symptoms of PTSD | 3.33 (0.73) |
| 2. | Helping me learn about treatments for PTSD | 2.96 (0.92) |
| 3. | Helping me find effective ways of managing my symptoms | 3.33 (0.73) |
| 4. | Helping me feel more comfortable in seeking support | 3.44 (0.90) |
| 5. | Helping me feel there is something I can do about my PTSD | 3.42 (0.70) |
| 6. | Helping me track my symptoms | 3.11 (0.90) |
| 7. | Helping me know when I’m doing better or when I’m doing worse | 3.04 (0.81) |
| 8. | Increasing my access to additional resources | 3.22 (1.09) |
| 9. | Providing practical solutions to the problems I experience | 3.22 (1.01) |
| 10. | Helping me overcome the stigma of seeking mental health services | 3.30 (0.95) |
| 11. | Helping me better understand what I have been experiencing | 3.41 (0.88) |
| 12. | Enhancing my knowledge of PTSD | 3.44 (0.70) |
| 13. | Helping clarify some of the myths about PTSD | 3.07 (1.03) |
| 14. | Providing a way for me to talk about what I have been experiencing | 3.67 (0.48) |
| 15. | Overall how satisfied are you with the PTSD Coach? | 3.62 (0.75) |

*Notes. Helpfulness and satisfaction ratings: 0 = not at all; 1 = slightly; 2 = moderately; 3 = very; 4 = extremely.*

*Supplementary Table 2. Self-Efficacy Managing PTSD Symptoms (n = 27).*

| **Item** | **Item** | **Mean (SD)** |
| --- | --- | --- |
| 1. | I can handle situations that remind me of the trauma? | 67.03 (21.09) |
| 2. | I can manage the stress of situations that remind me in some way of the trauma. | 71.86 (19.42) |
| 3. | I can feel more connected to people. | 71.85 (26.89) |
| 4. | I can feel more connected to reality. | 75.19 (22.76) |
| 5. | I can make myself feel less sad or hopeless. | 65.56 (29.80) |
| 6. | I can make myself feel less worried or anxious. | 68.89 (27.36) |
| 7. | I can control my anger. | 73.52 (22.69) |
| 8. | I can improve my sleep. | 78.52 (20.89) |
| 9. | I can use the skills from the PTSD Coach to manage my PTSD symptoms. | 81.86 (22.71) |
| 10. | I can reach out to friends or family when I need help. | 76.30 (24.52) |

*Notes. Ratings ranged from 0 to 100: 0 - 40 = cannot do at all; 50 - 80 = Moderately can do; 80 – 100 = Highly certain can do.*
