# Supplementary material for: A counsellor-supported ‘PTSD Coach’ intervention versus enhanced Treatment-as-Usual in a resource-constrained setting: A randomised controlled trial
Source: Glob Ment Health (Camb). 2024 Jan 3;11:e7. doi: 10.1017/gmh.2023.92 (PMC10808979; doi:10.1017/gmh.2023.92)
Supplement: Bröcker et al. supplementary material 2 — Bröcker et al. supplementary material [file S2054425123000924sup002.docx]

**Supplementary figures 1 - 4**

*Figure 1. PCL-5 Total Score: Interaction over time*

*Figure 2. DASS-Depression Total Score: Interaction over time*

*Figure 3.DASS-Anxiety Total Score: Interaction over time*

*Figure 4. DASS-Stress Total Score: Interaction over time*
