# Supplementary material for: A counsellor-supported ‘PTSD Coach’ intervention versus enhanced Treatment-as-Usual in a resource-constrained setting: A randomised controlled trial
Source: Glob Ment Health (Camb). 2024 Jan 3;11:e7. doi: 10.1017/gmh.2023.92 (PMC10808979; doi:10.1017/gmh.2023.92)
Supplement: Bröcker et al. supplementary material 3 — Bröcker et al. supplementary material [file S2054425123000924sup003.docx]

**APPENDIX A: Overview of Trial Measures**

| **Measure/Questionnaire** | **Description** |
| --- | --- |
| Demographic questionnaire | Captures data on age, gender, ethnicity, educational level, marital status, employment and annual income category, and past and current psychiatric care. |
| Global Psychotrauma Screen - **GPS** (Olff et al., 2020,2021) | A transdiagnostic 23-item (17 symptoms, five risk/protective factors, one functioning) screener for stress, trauma, and related reactions in the past month. Current data indicates it is a reliable and valid screening tool for trauma exposure and possible consequences (e.g., the likelihood of posttraumatic stress disorder (PTSD) (Frewen et al., 2021; Olff et al., 2020, 2021). |
| Mini-International Neuropsychiatric Interview - **MINI 7.0.0** (Sheehan & Lecrubier, 1998) | A structured clinician-administered diagnostic interview covering current and lifetime psychiatric disorders. Psychiatrists and clinicians initially developed it for the third edition of the Diagnostic and Statistical Manual of Mental Disorders (DSM -III) (Sheehan & Lecruibier, 1998). The MINI 7.0.0 aligns with the DSM, fifth edition (DSM-5). The MINI has been used in low to middle-income countries (LMICs), including South Africa (Ayazi et al., 2014; Suliman et al., 2014) (License agreement Study# N18/05/058 PACTR202108755066871). |
| Clinician-Administered PTSD Scale for DSM-5  - **CAPS-5** (past month)  (Weathers et al., 2013) | A 30-item structured clinician administered diagnostic interview considered as the gold standard in evaluating the frequency and severity of 20 posttraumatic stress disorder (PTSD) symptoms (Weathers et al., 2018). Previous versions have shown sound psychometric properties in over 200 studies (Weathers et al., 2001). The CAPS-5 is also deemed psychometrically sound and used effectively in South Africa (Suliman et al., 2014; Weathers et al., 2013, 2018). |
| Childhood Trauma Questionnaire (Short Form) - **CTQ-SF** (Bernstein et al., 1998) | A 28-item, retrospective, self-report questionnaire used to measure abuse (emotional, physical, sexual) and neglect (emotional and physical) before the age of 18 (Bernstein & Fink, 1998). The questionnaire has shown excellent validity for the full scale and acceptable to excellent validity for the subscales (Aloba et al., 2020; Scher et al., 2001). The CTQ-SF has evidenced as psychometrically sound when applied in South Africa (Roomaney et al., 2021). |
| Life Events Checklist for DSM-5 – **LEC-5**  (Weathers et al., 2013) | A 17-item self-report measure that screen for lifetime traumatic event exposure. The original LEC demonstrated convergent validity with other measures evaluating trauma exposure levels (Gray et al., 2004). Psychometric properties are currently unavailable for the newer LEC-5; however, similar psychometric properties are expected (Weathers et al., 2013). The LEC-5 has been evidenced as psychometrically sound when applied in South Africa (Stevenson et al., 2023). |
| PTSD Checklist for DSM-5 - **PCL-5** (Wortmann et al., 2016) | A 20-item self-report assessing 20 PTSD symptoms used to screen, provisionally diagnose, or monitor self-reported PTSD symptom severity. The PCL-5 assesses how much someone is bothered by the PTSD symptoms over the past month. As expected, the PCL-5 is psychometrically sound (Roberts et al., 2021; Wortmann et al., 2016). This measure has been evidenced as psychometrically sound when applied in South Africa (Kagee et al., 2022). |
| Depression, Anxiety and Stress Scale 21 - **DASS-21**  (Henry & Crawford, 2004) | Developed from the lengthier 42-item measure, this shortened 21-item version has shown adequate reliability and validity in both clinical and non-clinical populations, as well as when used in non-English speaking cultures (Henry & Crawford, 2004; Jun et al., 2018; Lovibond, 1998; Vignola et al., 2013). It consists of three subscales evaluating self-reported depression, anxiety, and stress symptoms. The DASS-21 has been successfully applied with sound psychometric properties in South Africa (Tsabedze et al., 2021; Tshabalala et al., 2019). |
| Resilience evaluation scale – **RES** (van der Meer et al., 2018) | A 9-item self-report measure that is freely available, brief, with sound psychometric properties (van der Meer et al., 2018). This scale identifies and measures psychological resilience post personal adversities faced by individuals. It has shown sound psychometric properties in other studies (Qing et al., 2022). |
| Multidimensional Scale of Perceived Social Support - **MSPSS** (Zimet et al., 1990) | A 12-item inventory that measures perceived adequacy of social support from three sources: family, friends and significant other (Zimet et al., 1990). The measure has sound psychometric properties (Zimet et al., 1988). It has been evidenced as psychometrically sound when applied in South Africa (Bruwer et al., 2008; Myint & Mash, 2008; Ogunbajo et al., 2020). |
| Alcohol  Use Disorders Identification Test - **AUDIT**  (Saunderset al., 1993) | A 10-item self-report questionnaire used to identify harmful alcohol use. This tool was developed by the World Health Organisation (WHO) and has good reliability and validity in diverse contexts (Bohnet al., 1995). It has been applied successfully in South Africa (Macleod et al., 2021; Ndungu et al., 2020; Stoloff et al., 2013). |
| Drug Use Disorders Identifications Test - **DUDIT**  (Matuszka et al., 2013) | A 11-item self-report questionnaire used to identify hazardous substance use. Developed by the WHO to use in parallel with the AUDIT and has good validity and reliability in both inpatient and outpatient settings (Matuszka et al., 2014; Voluse, et al., 2012). It has been applied successfully in South Africa (Stoloff et al., 2013; Vythilingum et al., 2012). |
| **Perceived helpfulness of the PTSD Coach app**  (Kuhn et al., 2017) | A 15-item self-report scale used with permission from the developer Dr E. Kuhn (Kuhn, et al., 2017). The first 14 items of the scale evaluate the user's experience of the PTSD Coach mobile application (from 0 = *"Not at all helpful"* to 4 = *"Extremely helpful."* The last item evaluates the user's overall satisfaction with the app (from 0 = *"Not at all satisfied"* to 4 = *"Extremely satisfied."* It was used successfully in non-English populations (Cernvall et al., 2018; Hensler et al., 2022). To our knowledge this scale has not been applied in South Africa apart from during pilot testing, and the 14 items measuring the perceived helpfulness of the app demonstrated high internal consistency (Cronbach's α = 0.93) (Bröcker et al., 2022). |
| **PTSD Coach self-efficacy scale**  (Kuhn et al., 2017) | A 10-item self-report scale used with permission from the developer Dr E. Kuhn (Kuhn et al., 2017). This scale evaluates the person's degree of confidence in managing their PTSD-related symptoms. Responses are based on a scale from 0 – 100, with 0 = *"Cannot do at all"*, 50 = *"Moderately can do"* and 100 = *"Highly certain can do."* To our knowledge this scale has not been applied in South Africa apart from during pilot testing and demonstrated good internal consistency (Cronbach's α = 0.84) in our sample. |

**References**

Aloba, O., Opakunle, T. & Ogunrinu, O. (2020). Childhood Trauma Questionnaire-Short Form (CTQ-SF): Dimensionality, validity, reliability and gender invariance among Nigerian adolescents. *Child Abuse & Neglect.* 101, 104357. <http://doi:10.1016/j.chiabu.2020.104357>.

Ayazi, T., Lien, L., Eide, A., Swartz, L. & Hauff, E. (2014). Association between exposure to traumatic events and anxiety disorders in a post-conflict setting: a cross-sectional community study in South Sudan. *BMC Psychiatry*, *14*(6). <http://doi:10.1186/1471-244X-14-6>. PMID: 24410951.

Bernstein, D. P., Stein, J. A., Newcomb, M. D., Walker, E., Pogge, D., Ahluvalia, T., Stokes, J., Handelsman, L., Medrano, M., Desmond, D., & Zule, W. (1995). Development and validation of a brief screening version of the Childhood Trauma Questionnaire. *Child Abuse & Neglect*, 27(2), 169-90. <http://doi:10.1016/s0145-2134(02)00541-0>.

Bohn, M. J., Babor, T. F., & Kranzler, H. R. (1995). The Alcohol Use Disorders Identification Test (AUDIT): validation of a screening instrument for use in medical settings. *Journal of Studies on Alcohol and Drugs*. *56*(4), 423-32. <http://doi:10.15288/jsa.1995.56.423>.

Bröcker, E., Olff, M., Suliman, S., Kidd, M., Mqaisi, B., Greyvenstein, L., Kilian, S., & Seedat S. (2022). A clinician-monitored 'PTSD Coach' intervention: findings from two pilot feasibility and acceptability studies in a resource-constrained setting. *European Journal of Psychotraumatology, 13(2)*: 2107359. <http://doi:10.1080/20008066.2022.2107359>.

Bruwer, B., Emsley, R., Kidd, M., Lochner, C., & Seedat S. (2008). Psychometric properties of the Multidimensional Scale of Perceived Social Support in youth. *Comprehensive Psychiatry, 49*(2), 195-201. <http://doi:10.1016/j.comppsych.2007.09.002>.

Cernvall, M., Sveen, J., Bergh Johannesson, K., & Arnberg, F. (2018). A pilot study of user satisfaction and perceived helpfulness of the Swedish version of the mobile app PTSD Coach. *European Journal of Psychotraumatology, 9*(Suppl 1): 1472990. <http://doi:10.1080/20008198.2018.1472990>.

Frewen P, McPhail I, Schnyder U, Oe M, Olff M. (2021). Global Psychotrauma Screen (GPS): psychometric properties in two Internet-based studies. *European Journal of Psychotraumatology, 12*(1): 1881725. <http://doi:10.1080/20008198.2021.1881725>. PMID: 34992750; PMCID: PMC8725737.

Gray, M., Litz, B., Hsu, J., & Lombardo, T. (2004). Psychometric properties of the Life Events Checklist. *Assessment, 11,* 330-341. <http://doi:10.1177/1073191104269954>.

Henry, J. D. & Crawford, J. R. (2005). The short-form version of the Depression Anxiety Stress Scales (DASS-21): construct validity and normative data in a large non-clinical sample. *British Journal of Clinical Psychology*, *44*(Pt 2), 227-239. <http://doi:10.1348/014466505X29657>.

Hensler, I., Sveen, J., Cernvall, M., & Arnberg, F. K. (2022). Efficacy, Benefits, and Harms of a Self-management App in a Swedish Trauma-Exposed Community Sample (PTSD Coach): Randomized Controlled Trial. *Journal of Medical Internet Research, 24*(3): e31419. <http://doi:10.2196/31419>.

Jun, D., Johnston, V., Kim, J. M., & O'Leary, S. (2018). Cross-cultural adaptation and validation of the Depression, Anxiety and Stress Scale-21 (DASS-21) in the Korean working population. *Work*, *59*(1):93-102. <http://doi:10.3233/WOR-172661>.

Kagee, A., Bantjes, J., Saal, W. & Sterley, A. (2022). Predicting Posttraumatic Stress Disorder Caseness Using the PTSD Checklist for DSM-5 Among Patients Receiving Care for HIV. *Journal of Traumatic Stress, 35*(1), 13-21. <http://doi:10.1002/jts.22654>.

Kuhn, E., Kanuri, N., Hoffman, J. E., Garvert, D. W., Ruzek, J. I., & Taylor, C.B. (2017). A randomized controlled trial of a smartphone app for posttraumatic stress disorder symptoms. *Journal of Consulting and Clinical Psychology*, *85*(3), 267-273. <http://doi:10.1037/ccp0000163>.

Lovibond, P. F. (1998). Long-term stability of depression, anxiety, and stress syndromes. *Journal of Abnormal Psychology, 107*(3), 520-526. <http://doi:10.1037//0021-843x.107.3.520>.

Macleod, C. I., Young, C., & Molokoe, K. (2021). Alcohol use during pregnancy: prevalence and patterns in selected Buffalo City areas, South Africa. *African Journal of Reproductive Health, 25*(1), 114-121. <http://doi:10.29063/ajrh2021/v25i1.13>.

Matuszka, B., Bácskai, E., Berman, A. H., Czobor, P., Sinadinovic, K., & Gerevich J. (2014). Psychometric characteristics of the Drug Use Disorders Identification Test (DUDIT) and the Drug Use Disorders Identification Test-Extended (DUDIT-E) among young drug users in Hungary. *International Journal of Behavioral Medicine, 21*(3), 547-55. <http://doi:10.1007/s12529-013-9336-8>.

Myint,T, & Mash B. (2008). Coping strategies and social support after receiving HIV-positive results at a South African district hospital. *South African Medical Journal, 98*(4), 276-278. PMID: 18637635.

Ndungu, J., Washington, L., Willan, S., Ramsoomar, L., Ngcobo-Sithole, M., & Gibbs, A. (2020). Risk factors for alcohol and drug misuse amongst young women in informal settlements in Durban, South Africa. *Global Public Health, 15*(9), 1322-1336. <http://doi:10.1080/17441692.2020.1775866>.

Ogunbajo, A., Iwuagwu, S., Williams, R., Biello, K. B., Kahler, C. W., Sandfort, T. G. M., & Mimiaga, M. J. (2020). Validation of depressive symptoms, social support, and minority stress scales among gay, bisexual, and other men who have with men (GBMSM) in Nigeria, Africa: a mixed methods approach. *BMC Public Health, 20*(1): 1023. <http://doi:10.1186/s12889-020-09127-0>.

Olff, M., Bakker, A., Frewen, P., Aakvaag, H., Ajdukovic, D., Brewer, D., Elmore Borbon, D. L., Cloitre, M., Hyland, P., Kassam-Adams, N., Knefel, M., Lanza, J. A., Lueger-Schuster, B., Nickerson, A., Oe, M., Pfaltz, M. C., Salgado, C., Seedat, S., Wagner, A., Schnyder, U., & Global Collaboration on Traumatic Stress (GC-TS) (2020). Screening for consequences of trauma – an update on the global collaboration on traumatic stress. *European Journal of Psychotraumatology*, 11: 1. <http://doi:10.1080/20008198.2020.1752504>.

Olff, M., Primasari, I., Qing, Y., Coimbra, B. M., Hovnanyan, A., Grace, E., Williamson, R. E., & Hoeboer, C. M; GPS-CCC Consortium. Mental health responses to COVID-19 around the world. (2021). *European Journal of Psychotraumatology,12*(1): 1929754. <http://doi:10.1080/20008198.2021.1929754>.

Qing Y., Bakker, A., van der Meer, C. A. I., Te Brake, H., & Olff, M. (2022). Assessing psychological resilience: translation and validation of the Chinese version of the resilience evaluation scale (RES). *European Journal of Psychotraumatology, 13*(2): 2133358. <http://doi:10.1080/20008066.2022.2133358>.

Roberts, N. P., Kitchiner, N. J., Lewis, C. E., Downes, A. J., & Bisson, J. I. (2021). Psychometric properties of the PTSD Checklist for DSM-5 in a sample of trauma exposed mental health service users. *European Journal of Psychotraumatology,12*(1): 1863578. <http://doi:10.1080/20008198.2020.1863578>.

Roomaney, A. A., Womersley, J. S., Swart, P. C., Spies, G., Seedat, S., & Hemmings, S. M. J. (2021). Childhood trauma and genetic variation in the DAT 40-bp VNTR contribute to HIV-associated neurocognitive disorders. *IBRO Neuroscience Reports, 12*, 45-54. <http://doi:10.1016/j.ibneur.2021.12.003>.

Scher, C. D., Stein, M. B., Asmundson, G. J., McCreary, D. R., & Forde, D. R. (2001). The childhood trauma questionnaire in a community sample: psychometric properties and normative data. *Journal of Traumatic Stress, 14*(4), 843-57. <http://doi:10.1023/A:1013058625719>.

Sheehan, D. V., Lecrubier, Y., Harnett-Sheehan, K., Amorim, P., Janavs, J., Weiller, E., Hergueta, T., Baker, R. & Dunbar, G. (1998). The Mini International Neuropsychiatric Interview (M.I.N.I.): The Development and Validation of a Structured Diagnostic Psychiatric Interview. *Journal of Clinical Psychiatry, 59*(Suppl 20), 22-33. PMID: 9881538

Stevenson, A., Beltran, M., Misra, S., Ametaj, A. A., Bronkhorst, A., Gelaye, B., Koenen, K. C., Pretorius, A., Stein, D. J., & Zingela, Z. (2023). Trauma exposure and psychometric properties of the life events checklist among adults in South Africa. *European Journal of Psychotraumatology, 14*(1): 2172257. <http://doi:10.1080/20008066.2023.2172257>.

Stoloff, K., Joska, J. A., Feast, D., De Swardt, G., Hugo, J., Struthers, H., McIntyre, J., & Rebe, K. (2013). A description of common mental disorders in men who have sex with men (MSM) referred for assessment and intervention at an MSM clinic in Cape Town, South Africa. *AIDS and Behavior, 17* (Suppl 1): S77-81. <http://doi:10.1007/s10461-013-0430-3>.

Suliman, S., Stein, D. J., & Seedat, S. (2014). Clinical and neuropsychological predictors of posttraumatic stress disorder. *Medicine*, *93*(22): e113. <http://doi:10.1097/MD.0000000000000113>.

Tsabedze, N., Kinsey, J.H., Mpanya, D., Mogashoa, V., Klug, E., & Manga, P. (2021). The prevalence of depression, stress and anxiety symptoms in patients with chronic heart failure. *International Journal of Mental Health Systems, 15*(1): 44. <http://doi:10.1186/s13033-021-00467-x>.

Tshabalala, S. J., Tomita, A., & Ramlall, S. (2019). Depression, anxiety and stress symptoms in patients presenting with dyspepsia at a regional hospital in KwaZulu-Natal province. *South African Journal of Psychiatry, 25*: a1382. <http://doi:10.4102/sajpsychiatry.v25i0.1382>.

van der Meer, C. A. I., Te Brake, H., van der Aa, N., Dashtgard, P., Bakker, A., & Olff M. (2018). Assessing Psychological Resilience: Development and Psychometric Properties of the English and Dutch Version of the Resilience Evaluation Scale (RES). *Frontiers Psychiatry, ;9*: 169. <http://doi:10.3389/fpsyt.2018.00169>.

Vignola, R. C. & Tucci, A. M. (2014). Adaptation and validation of the depression, anxiety and stress scale (DASS) to Brazilian Portuguese. *Journal of Affective Disorders, 155*: 104-9. <http://doi:10.1016/j.jad.2013.10.031>.

Voluse, A. C., Gioia, C. J., Sobell, L. C., Dum, M., Sobell, M. B., & Simco, E. R. (2012). Psychometric properties of the Drug Use Disorders Identification Test (DUDIT) with substance abusers in outpatient and residential treatment. *Addictive Behaviors, 37*(1), 36-41. <http://doi:10.1016/j.addbeh.2011.07.030>.

Vythilingum, B., Roos, A., Faure, S. C., Geerts, L., & Stein, D. J. (2012). Risk factors for substance use in pregnant women in South Africa. *South African Medical Journal, 102*(11 Pt 1), 851-854. <http://doi:10.7196/samj.5019>.

Weathers, F. W., Blake, D. D., Schnurr, P. P., Kaloupek, D. G., Marx, B. P., & Keane, T. M. (2013). *The Clinician-Administered PTSD Scale for DSM-5 (CAPS-5)*. [Assessment] Available from <https://www.ptsd.va.gov/professional/assessment/adult-int/caps.asp>.

Weathers, F. W., Blake, D. D., Schnurr, P. P., Kaloupek, D. G., Marx, B. P., & Keane, T. M. (2013). The Life Events Checklist for DSM-5 (LEC-5). Instrument available from the National Center for PTSD at [www.ptsd.va.gov](http://www.ptsd.va.gov)

Weathers, F. W., Keane, T. M., & Davidson, J. R. (2001). Clinician-administered PTSD scale: a review of the first ten years of research. *Depression and Anxiety, 13*(3), 132-156. <http://doi:10.1002/da.1029>.

Weathers, F., Bovin, M., Lee, D., Sloan, D., Schnurr, P., Kaloupek, D., . . . Marx, B. (2018). The Clinician-administered PTSD scale for DSM-5 (CAPS-5): Development and initial psychometric evaluation in military veterans. *Psychological Assessment, 30*(3), 383–395. <http://doi:10.1037/pas0000486>.

Wortmann, J. H., Jordan, A. H., Weathers, F. W., Resick, P. A., Dondanville, K. A., Hall-Clark, B., Foa, E. B., Young-McCaughan, S., Yarvis, J. S., Hembree, E. A., Mintz, J., Peterson, A. L., & Litz, B. T. (2016). Psychometric analysis of the PTSD Checklist-5 (PCL-5) among treatment-seeking military service members*. Psychological Assessment, 28*(11), 1392-1403. <http://doi:10.1037/pas0000260>.

Zimet, G. D., Powell, S. S., Farley, G. K., Werkman, S., & Berkoff, K. A. (1990). Psychometric characteristics of the Multidimensional Scale of Perceived Social Support. *Journal of Personality Assessment, 55*(3-4), 610-617. <http://doi:10.1080/00223891.1990.9674095>.
